# Characterization of the genomic landscape of canine diffuse large B-cell lymphoma reveals recurrent H3K27M mutations linked to progression-free survival

**DOI:** 10.1038/s41598-025-89245-0

**Published:** 2025-02-08

**Authors:** Anna Darlene van der Heiden, Raphaela Pensch, Sophie Agger, Heather L. Gardner, William Hendricks, Victoria Zismann, Shukmei Wong, Natalia Briones, Bryce Turner, Karin Forsberg-Nilsson, Cheryl London, Kerstin Lindblad-Toh, Maja Louise Arendt

**Affiliations:** 1 Department of Medical Biochemistry and Microbiology, Uppsala University, Uppsala, Sweden; 2 SciLifeLab, Uppsala University, Uppsala, Sweden; 3 Division of Integrated Cancer Genomics, Translational Genomics Research Institute (TGen), Phoenix, AZ US; 4 Department of Veterinary Clinical Sciences, University of Copenhagen, Copenhagen, Denmark; 5 Department of Immunology, Genetics and Pathology, Uppsala University, Uppsala, Sweden; 6 Cummings School of Veterinary Medicine, Tufts University, North Grafton, MA United States of America; 7 Broad Institute of MIT and Harvard, Cambridge, MA United States of America

**Keywords:** Dog, Canine, Diffuse large B-cell lymphoma, Prognosis, Genomics, Oncogenetics, Lymphoma, Oncology, Cancer genomics, Cancer models

## Abstract

Diffuse large B-cell lymphoma (DLBCL) is an aggressive hematopoietic neoplasm that affects humans as well as dogs. While previous studies on canine DLBCL (cDLBCL) have significantly advanced our understanding of the disease, the majority of this research has relied on whole-exome sequencing, which is limited in its ability to detect copy number aberrations and other genomic changes beyond coding regions. Furthermore, many of these studies lack sufficient clinical follow-up data, making it difficult to draw meaningful associations between genetic variants and patient outcomes. Our study aimed to characterize the mutational landscape of cDLBCL using whole-genome sequencing of matched tumor-normal samples obtained from a cohort of 43 dogs previously enrolled in a clinical trial for which longitudinal follow-up was available. We focused on identifying genes that were significantly or recurrently mutated with coding point mutations, copy number aberrations, and their associations with patient outcomes. We identified 26 recurrently mutated genes, 18 copy number gains, and 8 copy number losses. Consistent with prior studies, the most commonly mutated genes included *TRAF3*,* FBXW7*,* POT1*,* TP53*,* SETD2*,* DDX3X* and *TBL1XR1.* The most prominent copy number gain occurred on chromosome 13, overlapping key oncogenes such as *MYC* and *KIT*, while the most frequent deletion was a focal loss on chromosome 26, encompassing *IGL*,* PRAME*,* GNAZ*,* RAB36*,* RSPH14*, and *ZNF280B*. Notably, our set of recurrently mutated genes was significantly enriched with genes involved in epigenetic regulation. In particular, we identified hotspot mutations in two histone genes, *H3C8*, and *LOC119877878*, resulting in H3K27M alterations predicted to dysregulate gene expression. Finally, a survival analysis revealed that H3K27M mutations in *H3C8* were associated with increased hazard ratios for progression-free survival. No copy number aberrations were associated with survival. These findings underscore the critical role of epigenetic dysregulation in cDLBCL and affirm the dog as a relevant large animal model for interrogating the biological activity of novel histone-modifying treatment strategies.

**Supplementary Information:**

The online version contains supplementary material available at 10.1038/s41598-025-89245-0.

## Introduction

Lymphoma occurs frequently in dogs, representing over 80% of all hematopoietic neoplasia and up to 25% of all canine malignancies^1–4^. B-cell lymphoma (BCL), in particular diffuse large B cell lymphoma (DLBCL), is the most common subtype in dogs with treatment typically consisting of multi-agent chemotherapy (e.g., CHOP-based protocols incorporating cyclophosphamide, doxorubicin, vincristine, and corticosteroids). While most dogs with DLBCL undergo a complete remission following treatment with a CHOP based chemotherapy protocol, the reported median survival time after initiation of treatment ranges from only 10–14 months due to development of resistance to therapy^5–7^. In human patients, DLBCL is treated with CHOP based chemotherapy in combination with rituximab, a chimeric monoclonal antibody that targets CD20 expressed on the surface of B-cells (R-CHOP)^8^. With the addition of rituximab to CHOP, the overall 5-year and 10-year survivorship has improved to 80% and 70%, respectively^9–12^. However, several factors influence outcome including tumor genomic landscape, age, performance status and co-morbidities, among others, resulting in a range of survival at 10 years from 30–90%^12^.

Building off of the human experience, several researchers have interrogated the mutational landscape of canine DLBCL (cDLBCL) to both define key cancer drivers as well as to better understand how such mutations may affect outcome in companion animal dogs. To date, multiple genes have been identified as recurrently mutated in cDLBCL, including *TRAF3*,* SETD2*,* POT1*,* TP53*, and *FBXW7*, with *TRAF3* consistently being the most frequently mutated gene and exhibiting mutation frequencies exceeding 50% in some studies^13–17^. When investigating the prognostic role of genetic aberrations, mutations in *TP53* have been linked to an adverse prognosis in at least two studies, while mutations in *SETD2* have also been suggested to contribute to shorter survival times^14,18^.

Although these studies have significantly advanced our understanding of cDLBCL, they have primarily relied on whole-exome sequencing (WES) data. While WES is a valuable tool due to its cost-effectiveness and efficiency, its focus on coding regions can be restrictive, making the detection of genomic mutations such as copy number aberrations (CNAs) less sensitive and precise compared to whole-genome sequencing (WGS)^19^. Furthermore, most prior studies lacked detailed clinical follow-up information, hindering the ability to identify significant correlations between genetic alterations and patient outcomes.

The objective of this study was to characterize the mutational landscape of cDLBCL using data from a highly curated cohort of patients enrolled in a longitudinal prospective clinical trial, focusing on both coding point mutations and copy number aberrations (CNAs) using WGS data. In addition, we wanted to determine whether specific genes or CNAs were associated with survival in this patient population.

## Results

### Study cohort

A total of 49 dogs, representing 29 different breeds, were included in this study. Among these breeds, the Labrador retriever (*n* = 6, 12.2%), pit bull (*n* = 5, 10.2%), beagle (*n* = 3, 6.1%), and golden retriever (*n* = 3, 6.1%) were the most common. Additionally, there were four mixed-breed dogs (8.2%), where specific details regarding the contributing breeds were not available. Sex distribution was well-balanced, with 23 (46.9%) females and 26 (53.1%) males. Only one female and two males were not neutered. At the time of diagnosis, the participants had a mean age of 8.0 years (range 2.5–13.1 years), and a mean weight of 26 kg (range 10.4–79.8 kg). Of the entire cohort, 47 dogs were diagnosed with cDLBCL based on both flow cytometry and lymph node biopsy with immunohistochemical staining for CD3 and CD20^20^, while the remaining two were diagnosed with small to intermediate B-cell lymphoma (Supplementary Table S1, Supplementary Fig. S1). All dogs were treated with 1E4-cIgGB, a canine monoclonal anti-CD20 antibody, along with varying combinations of doxorubicin and specific small molecular inhibitors under therapeutic evaluation (RV1001, KPT-9274, TAK-981) as previously described by Dittrich et al. (2023), and McLinden et al. (2024)^20,21^. This resulted in 6 treatment types henceforth referred to as *arms* (Supplementary Table S1, Supplementary Table S2).

### Mutational analysis

**Fig. 1:**
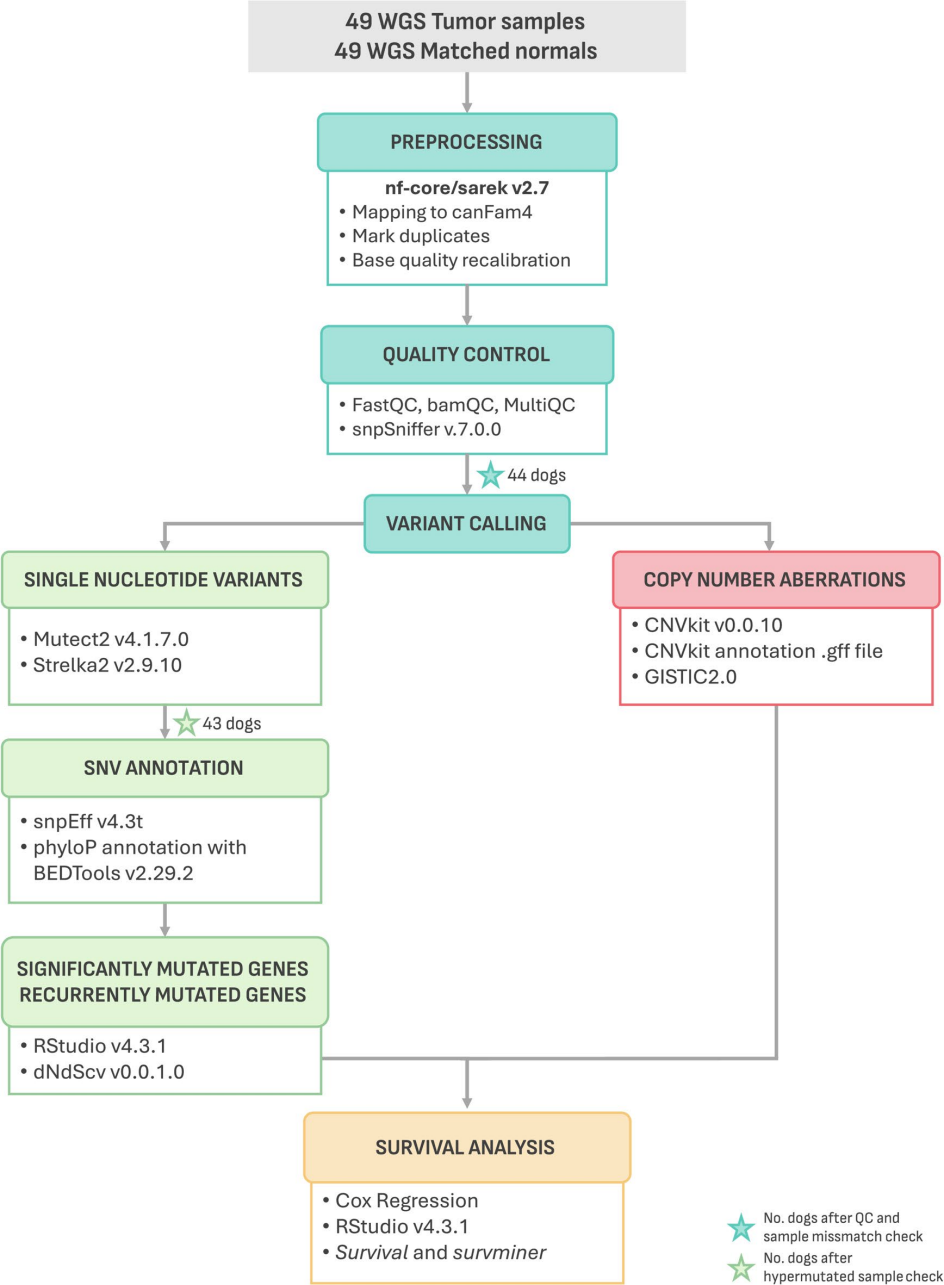
Workflow overview. Schematic representation of the analytical pipeline and bioinformatic tools utilized in this study. The initial canine cohort comprised 49 dogs, however, this number decreased as samples were excluded based on various quality control criteria. Star icons indicate the number of dogs remaining after each quality control step.

Matched tumor-normal whole-genome sequencing (WGS) data was generated from each dog. Raw sequencing data was aligned to the canFam4 (UU_Cfam_GSD_1.0)^22^ reference genome, resulting in a median coverage of 37x for normal samples (range 20.8–106.7, SD = 17.8) and 82x for tumor samples (range 52.7–143.9, SD = 19.0). Somatic variants were called using both Mutect2 and Strelka2, then annotated using snpEff for variant effect prediction and canine phyloP constraint scores from the Zoonomia project to assess evolutionary constraint (Fig. 1)^23–26^. To ensure the quality of the data, samples exhibiting tumor-normal mismatches, low sequencing quality, insufficient coverage (≤ 20x for normal samples and ≤ 50x for tumor samples), or abnormal mutation counts were excluded from the analysis. As a result, 6 dogs were removed from the initial cohort, leaving a final sample size of 43 patients (Fig. 1, Supplementary Fig. S2, Supplementary Table S1)^22–25^.

A total of 134,082 mutations were found across all 43 lymphoma samples. Of these 2,267 were present in the coding part of the genome, and 1,214 were found to be non-synonymous. Among these, 79.4% (*n* = 964) were missense variants, followed by 8.0% (*n* = 97) frameshift variants, and 5.7% (*n* = 69) stop gained variants. The complete breakdown of the different variant types is provided in Supplementary Table S3.

Tumor Mutational Burden (TMB), defined as the mean number of mutations per megabase (Mb) per sample, based on both non-coding and coding mutations ranged from 0.39 to 2.61 mutations per Mb, with a mean of 1.2 mutations per Mb, which is in alignment with previously published data regarding cDLBCL^14^.

### Significantly and recurrently mutated genes in cDLBCL

We identified a total of 953 genes with at least one non-synonymous mutation. Five of these were found to be under positive selection, exhibiting a significantly higher rate of non-synonymous mutations than expected by chance, as determined by dNdScv^27^ (global *q*-value ≤ 0.05). These Significantly Mutated Genes (SMGs) were *TRAF3*,* POT1*,* TBL1XR1*,* SETD2*, and *FBXW7* (Table 1, Supplementary Table S4).

**Table 1:** Significantly mutated genes (SMGs) in cDLBCL. No. Samples is the number of dogs with at least one mutation in a gene, and no. Mutations is the total number of somatic non-synonymous mutations found in a gene. The R package dNdScv (v.0.0.1.0), which is designed to detect genes under positive selection in cancer, was used to compute the global *p* and *q* values per gene. *P*-values were obtained utilizing the likelihood-ratio test, while *q*-values were obtained using Benjamini-Hodgberg’s multiple testing correction. Genes with a *q*-value ≤ 0.05 were considered to be under significant positive selection, and are shown in this table.

Gene name	No. samples	No. mutations	*p*-value	*q*-value
*TRAF3*	25	43	< 0.000	< 0.000
*POT1*	13	14	< 0.000	< 0.000
*SETD2*	10	12	< 0.000	0.003
*TBL1XR1*	8	11	< 0.000	0.004
*FBXW7*	13	19	< 0.000	0.022

Recurrently Mutated Genes (RMGs) were defined as genes found to be mutated in at least 5% (*n* = 3) of the samples. In total, 26 RMGs were identified. Notably, *TRAF3* was ranked highest, with mutations detected in 58.1% (*n* = 25) of the tumors, followed by *FBXW7* (30.2%, *n* = 13), *POT1* (30.2%, *n* = 13), *TP53* (25.6%, *n* = 11), *SETD2* (23.3%, *n* = 10), *TBL1XR1* (18.6%, *n* = 8), and *DDX3X* (18.6%, *n* = 8). These findings were consistent with previously published data regarding the mutational landscape of cDLBCL^13–17^. The complete list of RMGs can be found in Fig. 4, Supplementary Fig. S4, and Supplementary Table S5.

PhyloP scores of point mutations detected in the RMGs ranged from − 3.2 to 8.4, with a median of 6.1. Notably, variants in *TTN* and *SETD2* exhibited the highest levels of constraint, with all phyloP scores exceeding 8.3. *TTN* is a gene known for its large size (273 kb, canFam4) and the associated challenges of distinguishing driver mutations from passenger ones. However, the extremely high phyloP scores found in *TTN* mutations, suggesting that they occur at sites of high evolutionary constraint and critical importance, led us to retain this gene in our analysis (Supplementary Table S6, Supplementary Fig. S3).

**Fig. 2:**
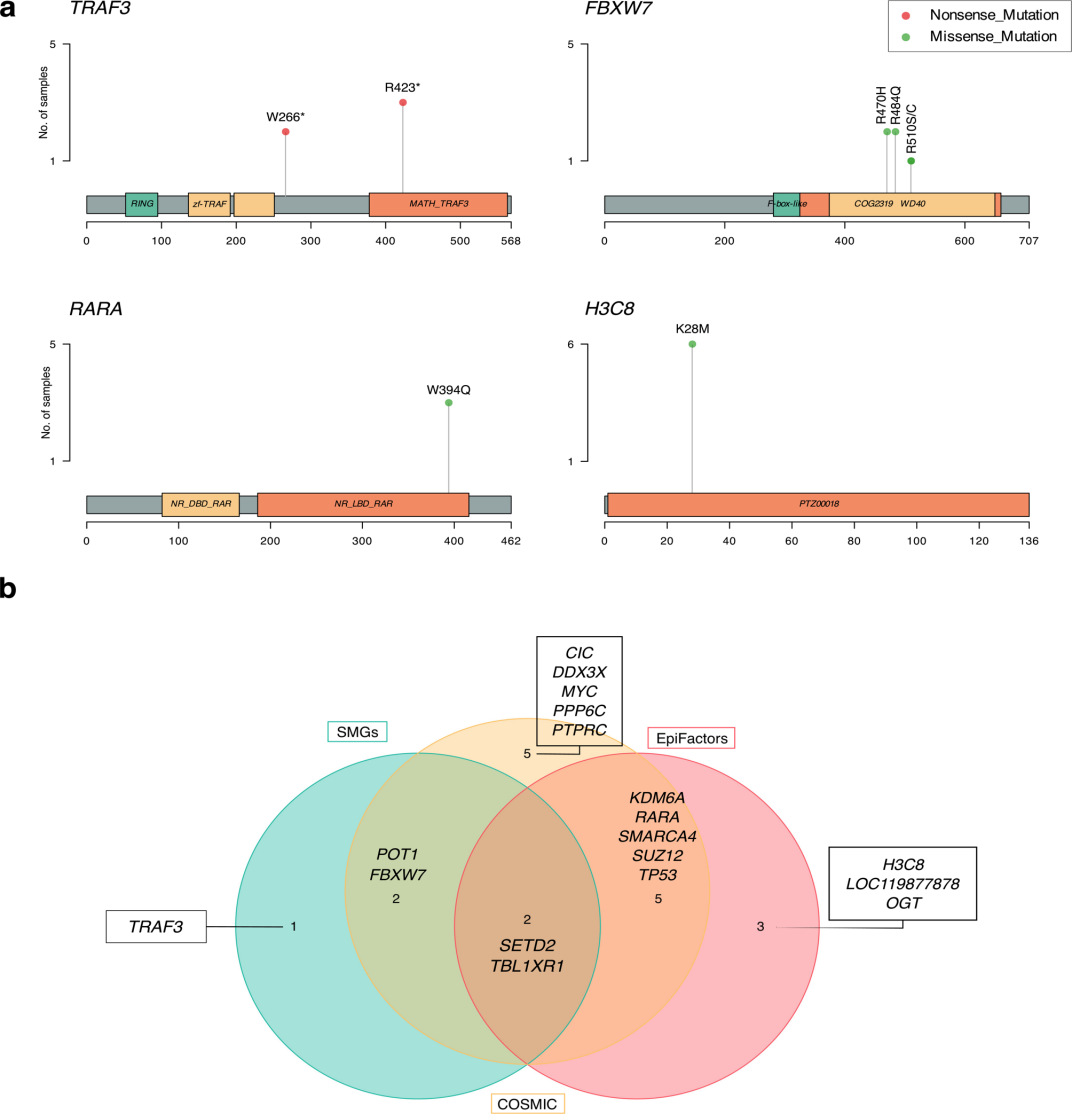
Recurrent mutations in RMGs and their representation in genomic databases. (**a**) Lollipop plots illustrating recurrently mutated positions in *TRAF3*,* FBXW7*,* RARA*, and *H3C8*. Each lollipop represents a specific mutation, with the stem height indicating mutation frequency. (**b**) Venn diagram showing the intersection of RMGs found in COSMIC (yellow) and EpiFactors (red) databases, as well as SMGs (blue).

Fifteen recurrently mutated positions were identified within the RMGs (Supplementary Fig. S5, Supplementary Fig. S6, Supplementary Table S7). Among the 43 variants detected in *TRAF3*, one base pair was mutated in three individuals and predicted to induce a premature stop codon (c.1267 C > T, p.R23*). A second position, also predicted to induce a premature stop codon, was found to be mutated in two individuals (c.798G > A, p.W266*). Another RMG with multiple recurrently mutated positions was *FBXW7*, which exhibited three variants detected in two individuals each, all of them predicted to be missense variants (c.1528 C > A, p.R510S; c.1528 C > T, p.R510C; c.1451G > A, p.R484Q; c.1409G > A, p.R470H). One of the most recurrently mutated positions was found in the RMG *RARA*, with three individuals carrying a p.1181G > A, p.R394Q missense mutation (Fig. 2).

Notably, all tumors with alterations in the histone genes *H3C8* and *LOC119877878* (a predicted histone 3.1 gene) carried the same H3 p.K28M mutation, specifically the substitution of lysine (K) with methionine (M) at position 28 of histone H3 (Fig. 2). This mutation is commonly referred to as H3K27M due to previous nomenclature practices that did not account for the first amino acid, thus we will use this term to refer to this mutation from this point onwards^28^. This type of mutation inhibits the methyltransferase activity of the Polycomb Repressive Complex 2 (PRC2), resulting in reduced H3K27 trimethylation and aberrant gene expression, and it has been implicated in tumor formation, maintenance, and poor prognosis in many cancers, including human pediatric high-grade gliomas^29–33^.

To investigate potential connections to human cancer, we cross-referenced our RMGs with the Catalogue of Somatic Mutations in Cancer (COSMIC) database and found 14 RMGs (53.8%) present in COSMIC^34^. These included *FBXW7*,* POT1*,* TP53*,* SETD2*,* DDX3X*,* TBL1XR1*,* KDM6A*,* MYC*,* RARA*,* SUZ12*,* PPP6C*,* PTPRC*,* CIC*, and *SMARCA4*, of which *TBL1XR1* and *MYC* in particular are listed as implicated in various types of human B-cell lymphoma (Fig. 2).

The EpiFactors database was used to identify which RMGs were classified as epigenetic factors, including histones, histone modifiers, chromatin remodelers, histone chaperones, protamines, protein cofactors forming complexes with epigenetic factors, DNA and RNA modifiers, and lncRNAs^35,36^. In total, 10 RMGs (38%) were classified as epigenetic factors, with two of them (*H3C8* and *LOC119877878*) identified as histone genes, and the remaining eight (*TP53*,* SETD2*,* TBL1XR1*,* SUZ12*,* RARA*,* KDM6A*,* OGT*, and *SMARCA4*) categorized as either epigenetic modulators or modifiers with histone modification functions. A Fisher’s exact test revealed that our RMGs list is indeed significantly enriched with epigenetic factors (*p* = 5.61e-8, CI 95% = [5.7–33.2], odds ratio 14.1). These results suggest that epigenetic dysregulation may play an important role in the development and progression of cDLBCL (Fig. 2).

To further explore functional implications of the identified RMGs, we conducted a pathway enrichment analysis. This revealed the pathways associated with chromatin-modifying enzymes and chromatin organization as the most significant (R-HSA-3247509 and R-HSA-4839726, FDR = 3.51e-5), with 8 genes (*SUZ12*,* H3C8*,* SETD2*,* TBL1XR1*,* OGT*,* SMARCA4*, and *KDM6A*) out of 254 annotated to these pathways.

### CNA analysis

**Fig. 3:**
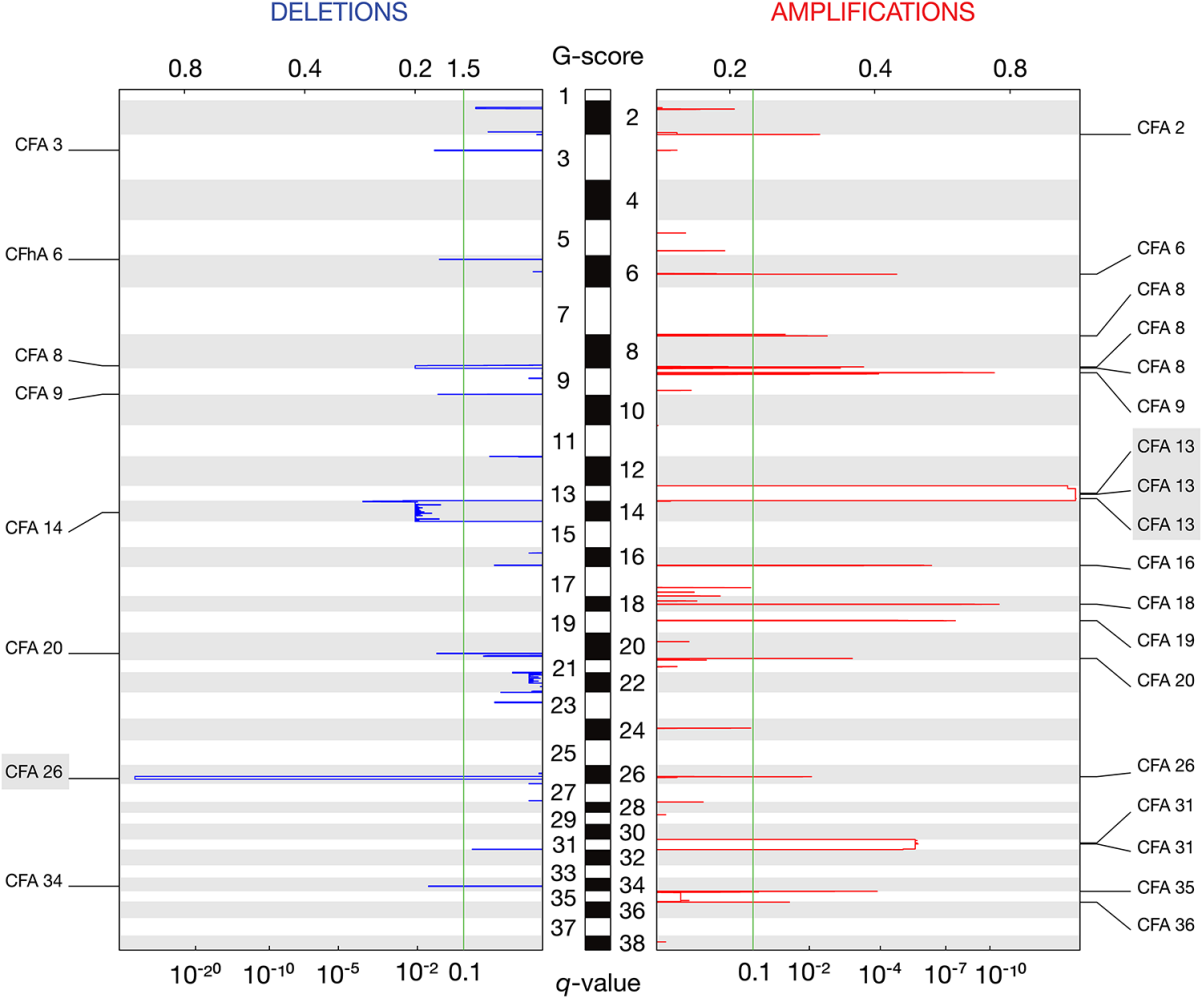
CNAs detected by GISTIC2.0. Deletion peaks are shown on the left in blue, while amplification peaks are displayed on the right in red. The central bar, alternating in black and white, represents all 38 canine autosomes. G-scores are indicated at the top of the plot, and *q*-values are shown at the bottom. The green line denotes the significance threshold (*q* = 0.10). CNAs with a G-score equal or greater than 1.0 are highlighted in grey.

**Fig. 4:**
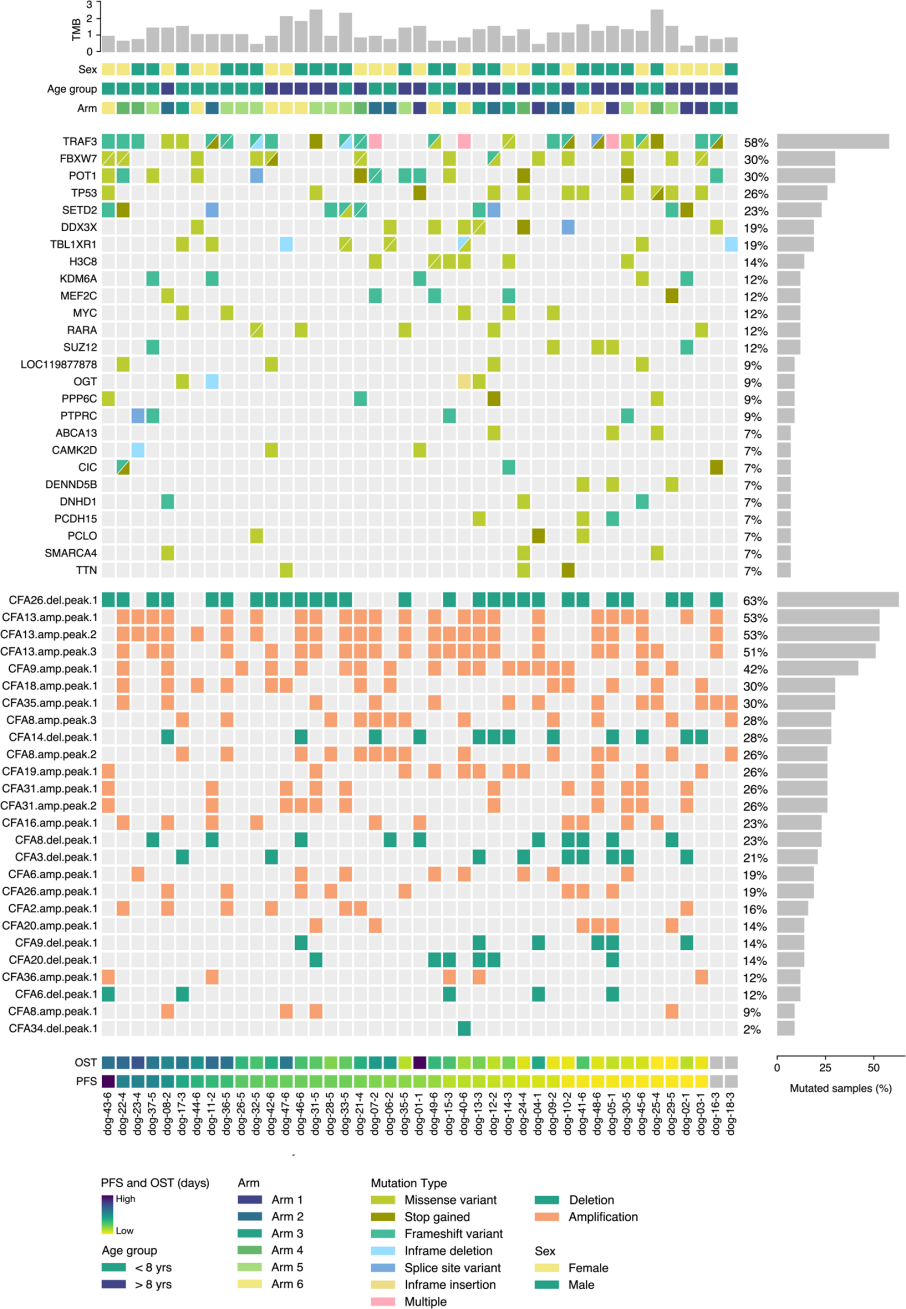
Mutational landscape in cDLBCL. Oncoplot showcasing the complete list of 26 RMGs (top) and signficant CNAs identified via GISTIC2.0 (bottom panel). Dog samples are arranged from left to right in descending order of PFS in days. The highest PFS is colored in dark purple, while the lowest is colored in yellow. OST uses the same color scheme. Unavailable survival data is denoted in grey.

Copy number aberration (CNA) analysis was carried out using CNVkit for copy number calling and GISTIC2.0 for significant CNA detection^37,38^. This revealed 26 significantly altered regions across the canine cohort, including 18 amplifications in canine chromosomes (CFA) 2, 6, 8, 9, 13, 16, 18, 19, 20, 26, 31, 35, and 36, as well as 8 deletions in CFA 3, 6, 8, 9, 14, 20, 26, and 34 (Figs. 3 and 4, Supplementary Table S8).

The most notable amplification was found in CFA 13 (53.5%, *n* = 23), with a G-score greater than 1.0, indicating its significance in size and frequency. This alteration has previously been reported in cDLBCL, and our data demonstrates that it typically spans the entirety of CFA13, resulting in copy number gains of several oncogenes such as *MYC* and *KIT.*^39,40^ The only significant deletion with a G-score greater than 1.0 was located in CFA 26 (62.8%, *n* = 27), overlapping several genes including *PRAME*,* GNAZ*,* RAB36*,* RSPH14*, and *ZNF280B*, as well as the immunoglobulin lambda locus (*IGL*) (Supplementary Fig. S7).

### Survival analysis

To evaluate the impact of the RMGs and CNAs on progression-free survival (PFS) and overall survival time (OST), a survival analysis was performed using Cox proportional-hazard regression. Due to missing PFS and OST data, two dogs had to be removed from the cohort, leaving a final sample size of 41 individuals.

We first conducted a univariate Cox regression analysis to identify which RMGs, CNAs, and clinical variables could potentially have an effect on PFS or OST. Due to our small sample size and the associated statistical challenges, we opted for an exploratory approach and used a relaxed *p-*value threshold of ≤ 0.10. Our results indicate that mutation in *DENND5B* (PFS HR = 8.9, 95% CI [2.2–36.6], *p* < 0.01; OST HR = 4.1, 95% CI [1.2–14.3], *p* = 0.03) and *ABCA13* (PFS HR = 5.1, 95% CI [1.4–18.2], *p* = 0.01; OST HR = 7.4, 95% CI [1.9–28.7], *p* < 0.01) were associated with an increase in hazard ratio (HR) for both PFS and OST, indicative of an unfavorable patient outcome. In contrast, mutations in *POT1* (PFS HR = 0.5, 95% CI [0.2–1.0], *p* = 0.05; OST HR = 0.4, 95% CI [0.2–1.0], *p* = 0.05) were associated with a decrease in HR for both PFS and OST, suggesting a favorable outcome. Furthermore, alterations in *H3C8* (HR = 2.4, 95% CI [0.9–6.2], *p* = 0.07), and *PCDH15* (HR = 4.0, 95% CI [1.1–14.1], *p* = 0.03) were exclusively linked with an increased HR for PFS. Lastly, mutations in *TP53* (HR = 2.0, 95% CI [0.9–4.5], *p* = 0.08), *DDX3X* (HR = 2.1, 95% CI [0.9–5.1], *p* = 0.09), and *CAMK2D* (HR = 0.15, 95% CI [0.0–1.2], *p* = 0.07) exhibited an association with elevated HR for OST without notable effects on PFS (Table 2, Supplementary Table S9).

**Table 2:** Univariate Cox regression results. Only variables with a *p*-value ≤ 0.10 in either PFS or OST are shown. Arm 1 was used as reference for treatment. Median age and TMB were used as cutoff for grouping age and TMB. RgC = regression coefficient, HR = hazard ratio, 95% CI = confidence intervals, *p* = *p*-value, FDR = false Discovery Rate/Benjamin Hochberg procedure.

Variable	Progression free survival (PFS)					Overall Survival Time (OST)				
	RgC	HR	95%CI	*p*	FDR	RgC	HR	95%CI	*p*	FDR
*Recurrently Mutated Genes (RMGs)*										
*POT1*	-0.78	0.46	0.21–0.98	0.05	0.23	-0.90	0.41	0.16–1.01	0.05	0.45
*TP53*	0.55	1.74	0.83–3.64	0.14	0.48	0.71	2.04	0.92–4.53	0.08	0.54
*DDX3X*	0.47	1.59	0.68–3.72	0.28	0.71	0.76	2.13	0.89–5.11	0.09	0.54
*H3C8*	0.87	2.38	0.92–6.16	0.07	0.29	0.55	1.72	0.64–4.68	0.28	0.79
*ABCA13*	1.62	5.06	1.41–18.2	0.01	0.16	2.00	7.38	1.9–28.7	< 0.01	0.24
*CAMK2D*	-0.50	0.61	0.19–2.0	0.41	0.71	-1.87	0.15	0.02–1.20	0.07	0.54
*DENND5B*	2.18	8.87	2.15–36.6	< 0.01	0.07	1.40	4.07	1.16–14.3	0.03	0.33
*PCDH15*	1.37	3.95	1.11–14.1	0.03	0.20	1.01	2.75	0.79–9.61	0.11	0.61
*Copy Number Aberrations (CNAs)*										
CFA19.amp.peak.1	0.20	1.22	0.57–2.63	0.61	0.84	0.93	2.53	1.11–5.77	0.03	0.33
CFA20.amp.peak.1	1.06	2.88	1.14–7.26	0.02	0.17	1.11	3.04	1.19–7.78	0.02	0.33
CFA3.del.peak.1	0.76	2.13	0.99–4.59	0.05	0.23	1.09	2.99	1.31–6.81	0.01	0.28
CFA9.del.peak.1	1.12	3.07	1.22–7.74	0.02	0.17	1.03	2.80	1.09–7.23	0.03	0.33
CFA14.del.peak.1	0.81	2.24	1.11–4.55	0.03	0.17	0.42	1.51	0.68–3.35	0.31	0.80
*Clinical variables*										
Treatment Arm 2	-1.43	0.24	0.07–0.81	0.02	0.17	-0.14	0.87	0.23–3.34	0.84	0.93
Treatment Arm 3	-1.36	0.26	0.07–1.01	0.05	0.23	0.04	1.04	0.22–4.88	0.96	0.97
Treatment Arm 4	-2.03	0.13	0.03–0.52	< 0.01	0.07	-0.65	0.52	0.12–2.36	0.40	0.87
Treatment Arm 5	-1.70	0.18	0.06–0.59	< 0.01	0.07	-0.06	0.94	0.25–3.49	0.92	0.97
Treatment Arm 6	-1.71	0.18	0.06–0.59	< 0.01	0.07	0.03	1.03	0.29–3.59	0.97	0.97
Age group (≥ 8 years)	0.80	2.22	1.12–4.45	0.02	0.23	0.60	1.83	0.87–3.86	0.11	0.62
TMB group (≥ 1.18)	0.56	1.75	0.91–3.36	0.10	0.36	0.55	1.74	0.83–3.65	0.14	0.61

A focal amplification in CFA 20 (PFS HR = 2.9, 95% CI [1.1–7.3], *p* = 0.02; OST HR = 3.0, 95% CI [1.2–7.8], *p* = 0.02), along with deletions in CFA 3 (PFS HR = 2.1, 95% CI [1.0–4.6], *p* = 0.05; OST HR = 3.0, 95% CI [1.3–6.8], *p* = 0.01) and CFA 9 (PFS HR = 3.1, 95% CI [1.2–7.7], *p* = 0.02; OST HR = 2.8, 95% CI [1.1–7.2], *p* = 0.03), were associated with an increased HR for both PFS and OST. A deletion spanning nearly the entirety of CFA 14 (HR = 2.2, 95% CI [1.1–4.6], *p* = 0.03) was exclusively linked to a decrease in PFS, while an amplification located in CFA 19 (HR = 2.5, 95% CI [1.1–5.8], *p* = 0.03) was associated with an unfavorable HR regarding OST (Table 2). Detailed information regarding the genes located within each amplification and deletion is provided in Supplementary Table S8.

When assessing the association between treatment arms and PFS, it was found that arms 2, 3, 4, 5, and 6 had better outcomes (*p* = 0.02, 0.05, < 0.01, < 0.01, and < 0.01 respectively) compared to arm 1, which is the only treatment course where doxorubicin wasn’t administered. Additionally, older age (≥ 8 years of age, *p* = 0.02) and higher TMB (≥ 1.18, *p =* 0.10) were associated with an increased HR for PFS. In contrast, no clinical variables showed any correlation with OST (Table 2, Supplementary Table S9).

It is important to mention that after adjusting for multiple testing using the Benjamin- Hochberg’s procedure with a traditional false discovery rate (FDR) threshold of ≤ 0.10, only *DENND5B* (FDR = 0.07) and treatment arms 4, 5, and 6 (FDR = 0.07, 0.07, 0.07) were significantly associated with PFS. No variable was significantly associated with OST after this correction (Table 2, Supplementary Table S9). Consequently, our previously described univariate Cox regression results, which used a *p-*value threshold of ≤ 0.10 to select variables of interest, should be interpreted as trends rather than definitive evidence of significant associations with patient outcome.

Given that OST can be affected by the owner’s decision to pursue additional treatment, and considering the lack of clinical variables associated with OST in the univariate analysis, we chose to focus on PFS for the next phase of our analysis.

To evaluate the potential influence of treatment arm, age group, TMB, and genetic aberrations on PFS, a multivariate Cox regression analysis was conducted including each of the variables that were suggested to be associated with survival in the univariate analysis (Concordance 0.77, Supplementary Table S10). This revealed that mutations in the histone gene *H3C8* (HR = 8.6, 95% CI = [2.3–32.1], *p* < 0.01) were linked to an increase in HR in relation to PFS, while treatment arms 3 (HR = 0.1, 95% CI = [0.0–1.5], *p* = 0.05) and 6 (HR = 0.2, 95% CI = [0.0–0.8], *p* = 0.03) were associated with lower HR. No CNA was found to be significantly associated with PFS.

## Discussion

In this study we characterize the mutational landscape of cDLBCL by investigating genetic aberrations in a cohort of 43 pet dogs treated as part of a clinical research trial. Our analysis not only validates prior findings but also provides novel insights, revealing previously unidentified potential candidates associated with patient outcome.

We identified a total of 26 RMGs, among which *TRAF3*,* TP53*,* POT1*,* FBXW7*,* SETD2*,* TBL1XR1*,* DDX3X*,* MEF2C*,* MYC*, and *KDM6A* have been consistently reported as recurrently mutated in canine B-cell lymphoma^13–17^. Additionally, *H3C8*,* RARA*,* SUZ12*,* CIC*,* ABCA13*, and *TTN* have been documented as frequently mutated in at least one publication^14^. These findings indicate that our results corroborate and align with existing published data on cDLBCL. In contrast, mutations in *PPP6C*,* LOC119877878*,* OGT*,* PTPRC*,* PCLO*,* SMARCA4*,* DNHD1*,* PCDH15*,* DENND5B*, and *CAMK2D*, have not been implicated in cDLBCL prior to this study, to our knowledge.

Notably, our RMGs list was significantly enriched with genes involved in epigenetic regulation, including two histone genes and eight genes associated with chromatin remodeling and histone modification. Moreover, chromatin modification and chromatin organization pathways ranked as our most significantly altered pathways. These results mirror what has been observed in human DLBCL (hDLBCL), where genetic aberrations disrupting chromatin remodeling and other epigenetic regulatory mechanisms are among the most frequent alterations and are considered a feature of the disease^41,42^.

Epigenetic aberrations play a crucial role in tumorigenesis by altering gene expression, granting tumors the advantage to proliferate and thrive. In hDLBCL, key epigenetic regulators such as *KMT2D*, *CREBBP*, and *EZH2* are frequently mutated^41–43^. These genes often serve as markers for specific hDLBCL subtypes; for instance, mutations in *EZH2* are particularly associated with the germinal center B-cell (GCB) subtype^43^. However, none of these genes were identified among our RMGs. This observation is consistent with other studies reporting lower frequencies of *EZH2* mutations in cDLBCL compared to in humans, and there is limited information regarding *KMT2D* and *CREBBP* mutations in cDLBCL^14^.

Though they share certain similarities, dogs and humans are two distinct species with unique biological characteristics. Research on cDLBCL has shown that while the specific genes affected may differ between species, the pathways involved are largely analogous^13,44^. For example, we observed mutations in *SUZ12* in 12% of our samples, this gene is a component of the PRC2 complex alongside *EZH2*^45^. Additionally, *KDM6A*, was also mutated at a rate of 12% in our cohort and interacts with *KMT2D*, as both are components of the COMPASS complex, which plays a crucial role in gene regulation through H3K4 trimethylation and H3K27 di- and tri- methylation^46,47^. Despite variations in mutation frequencies and affected genes across species, the significance of epigenetic dysregulation in cDLBCL is evident, underscoring its critical role in the disease.

Copy number aberrations also play an important role in cancer development and progression. Our analysis revealed several recurrent chromosomal aberrations in our cohort, with gain of CFA 13 and a focal deletion on CFA 26 being the most frequent. Broad gains on CFA 13, often spanning the entire chromosome, are well-established aberrations in canine BCL, and are known to affect critical oncogenes such as *MYC* and *KIT.*^39,40,48^ The genomic loss in CFA 26, comprising *PRAME*,* GNAZ*,* RAB36*,* RSPH14*,* ZNF280B*, and the immunoglobulin lambda locus (*IGL*), has also been identified in previous works^39,48^. This CNA closely resembles similar deletion events occurring at the human 22q11.22 locus. Although some studies have linked 22q11.22 deletions, particularly those involving the loss of *PRAME*, to tumor progression and poor prognosis in hDLBCL and other B-cell malignancies^49,50^, others argue that this deletion occurs as a consequence of normal *IGL* rearrangement during B-cell development and is likely not pathogenic^51,52^. Given the ongoing debate surrounding the significance of this deletion in human B-cell neoplasms, coupled with its similarity to our canine findings, we cannot definitively conclude whether the observed CFA 26 deletion is indeed contributing to the disease, highlighting the need for further investigation.

Our survival analysis suggests a potential association between mutations in *H3C8* and PFS. Interestingly, none of the previously identified prognostic genes, such as *TP53* and *SETD2*, were found to be associated with PFS in our study^14,18^. Several factors may explain this discrepancy, including sample size and difference in treatment protocols used between studies.

All canine tumors with alterations in *H3C8* invariably exhibited H3K27M mutations. This mutation was first reported by Giannuzzi et al.^14^ in canine lymphoma. Building on their work, our study not only confirms these previous findings but also provides evidence that this mutation may be associated with poor survival in cDLBCL. Though the functionality of this mutation has not been studied in detail across canine cancers, human studies have demonstrated that the H3K27M mutation causes significant epigenetic changes. Specifically, it leads to a global loss of H3K27 trimethylation through inhibition of PRC2 methyltransferase activity, resulting in impaired gene silencing and altered gene expression^30–33^. This mutation is frequently observed in pediatric brain tumors, notably pediatric high-grade glioma (pHGG) and diffuse midline glioma (DMG) and is associated with a worse prognosis in young patients^53,54^. Novel treatments targeting this specific mutation are being developed and tested. A prime example is the H3K27M-specific vaccine, or H3K27M-vac, which has been designed to treat patients with DMG. This vaccine has shown promise in human clinical trials, successfully inducing an immune response against H3K27M^+^ tumors.^55^ Epigenetic drugs have also proven effective for treatment. The histone demethylase inhibitor GSK-J4, for instance, is capable of increasing levels of trimethylated H3K27 and inhibiting tumor cell proliferation^56,57^.

Given our results regarding the H3K27M mutation, we hypothesize that similar mechanisms may be at play in cDLBCL, and that the presence of H3K27M mutations could confer a selective advantage to cancer cells through aberrant gene expression. Therefore, further investigation into this mutation and its relationship with survival outcomes in dogs diagnosed with cDLBCL is essential. Such research could lead to the potential incorporation of H3K27M as a biomarker in screening panels and the development of targeted therapies similar to the H3K27M-vac. Moreover, these insights suggest that dogs could serve as a relevant comparative model for evaluating the efficacy of epigenetic drugs, ultimately contributing to translational research that informs human studies.

Although our survival analysis provided valuable insights into potential candidate genes associated with PFS, it is important to acknowledge some limitations of our approach. Notably, the small sample size of our study reduces the statistical power of the analysis, which may affect the reliability of the results. Furthermore, during the initial stage of our investigation, we adopted a more exploratory approach by employing a *p*-value threshold of 0.10 instead of a more traditional FDR threshold. This should be considered when interpreting our findings, as it increases the likelihood of false positives. Nevertheless, the results from our multivariate analysis suggest that H3K27M mutations in *H3C8* warrant further research to validate their relevance.

In conclusion, our findings underscore the significance of epigenetic alterations in cDLBCL, particularly H3K27M mutations in the histone gene *H3C8*, which may be relevant for therapeutic research. However, additional studies are needed to evaluate their true impact on survival. Our results add to the existing knowledge on cDLBCL, contributing to a better understanding of the disease and potentially aiding comparative studies with humans, which may eventually support the development of treatments for both species.

## Methods

### Ethical statement

The animal protocol associated with this study was approved by the Tufts University Institutional Animal Care and Use Committee (IACUC, #G2017-110, #G2020-82). Owners of participant dogs provided written, signed informed consent to participate in this study. Documentation throughout the study was completed using REDCap. Disease was not induced in any dog, and no dogs were sacrificed for this study. Lymph node aspiration and biopsy are standard clinical procedures performed in dogs using mild sedation with dexmedetomidine and butorphanol, and lidocaine for local anesthesia. Dogs were provided with analgesics as necessary throughout the study to alleviate suffering as needed at the discretion of the attending clinician. All methods were carried out in accordance with relevant regulations and guidelines, including ARRIVE guidelines.

### Study design and clinical protocol

All dogs included in this study were part of a clinical trial conducted by Dittrich et al. (2023) and McLinden et al. (2024).20,21 Dogs were enrolled if they had a definitive diagnosis of B-cell lymphoma based on cytology and subsequent flow cytometric immunophenotypic confirmation of B-cell neoplasia, as well as a lymph node biopsy documenting DLBCL. All individuals were required to be at least one year of age, weigh ≥ 8 kg, and have at least two peripheral lymph nodes measuring ≥ 2 cm in diameter. Proper organ function was assessed through complete blood count, urinalysis, and serum biochemistry profile. Furthermore, pregnant or lactating dogs, as well as dogs with central nervous system involvement, autoimmune diseases, or any disease (such as cardiovascular, renal, or hepatic disease) that could reduce their tolerance to doxorubicin chemotherapy or interfere with the interpretation of the treatment effect, were excluded from the study

All dogs were treated with one of six different treatment protocols, also referred to as arms. Each arm consisted of a combination of 1E4-cIgGB, a canine monoclonal anti-CD20 antibody, and one or two small molecular inhibitors: RV1001 (Rhizen Pharmaceuticals AG, Basel, Switzerland), KPT-9274 (Karyopharm Therapeutics, Newton, USA), and TAK-981 (Takeda Pharmaceuticals, Tokyo, Japan). Doxorubicin was administered in all arms, with the exception of arm 1. The specific treatment protocols for each arm are described in Supplementary Table S2. Additional details regarding participant enrollment, eligibility criteria, and treatment protocols can be found in the published works of Dittrich et al. (2023) and McLinden et al. (2024)^20,21^.

### Sample collection and nucleic acid extraction

Peripheral blood or saliva representing constitutional DNA as well as tumor lymph node biopsy or needle aspirate representing tumor DNA were collected at enrollment and at disease progression. Tumor tissue samples, not exceeding 30 mg, were homogenized with QIAshredder (Qiagen), and the flow-through was used for nucleic acid isolation. DNA extraction was performed using either the AllPrep DNA/RNA Mini Kit (Qiagen) or DNeasy Blood and Tissue Kit (Qiagen) for constitutional blood and tumor samples, while the prepIT-L2P (DNAgenotek) was used for constitutional saliva samples. DNA quality and quantity were measured using NanoDrop spectrophotometer (ThermoFisher Scientific), TapeStation Genomic DNA Assay (Agilent Technologies), and Qubit Fluorometer 2.0 (ThermoFisher Scientific). Genomic DNA was stored at -20 °C until preparation of sequencing libraries was completed.

### Whole genome sequencing

Amplified or PCR-free whole genome sequencing libraries were prepared from 250 or 500ng DNA, respectively, using KAPA HyperPrep Kit (Roche) according to manufacturer’s protocol. Quality control was carried out using the Qubit Fluorometer 2.0 (ThermoFisher Scientific) and TapeStation D1000 assay. Sequencing libraries were pooled in equimolar amounts and sequenced on the NovaSeq 6000 platform (Illumina). Whole genome libraries were sequenced to a target of > 40/60x coverage normal/tumor. FASTQ files were generated using Illumina bcl2fastq2.

### Data preprocessing

Raw FASTQ files were mapped to the canine reference genome canFam4 (UU_Cfam_GSD_1.0)^22^ using the nf-core/sarek pipeline (v2.7) in combination with the workflow system Nextflow (v21.10.6)^58^. The nf-core/sarek pipeline, which was developed in accordance with the Genome Analysis Toolkit (GATK) Best Practices, includes read mapping (BWA-mem2 v.2.0), duplicate marking (GATK *MarkDuplicates* v.4.1.7.0), and base quality recalibration (GATK *BaseRecalibrator*, GATK *ApplyBQSR* v.4.1.7.0). Additionally, the pipeline creates a series of reports (FastQC, bamQC, and MultiQC), to assess the quality and reliability of the data.

Quality control of the resulting BAM files was carried out by manually inspecting the reports generated by the nf-core/sarek pipeline. Samples with a median coverage ≤ 20x for normal samples and ≤ 50x for tumor samples were excluded from downstream analysis. To ensure sample integrity and preventing mismatching, the genotype concordance checking tool snpSniffer (v.7.0.0) was used (https://github.com/tgen/snpSniffer). All tumor-normal sample pairs with a concordance ratio below 0.98 were considered mismatched and removed from the study, resulting in the removal of five samples.

### Somatic variant calling and annotation

Variant calling was performed using the *variant_calling* step of the nf-core/sarek pipeline (v2.7), employing GATK Mutect2 (v4.1.7.0) and Strelka2 (v2.9.10) as the primary callers^25,26,58^.

First, a Panel of Normals (PON) was generated by running Mutect2 in tumor-only mode on all normal samples, followed by using GATK (v4.1.4.1) *CreateSomaticPanelOfNormals* to construct the final PON^26^. For tumor somatic variant calling, both Mutect2 and Strelka2 were employed. During the Mutect2 variant calling process, the PON was applied to filter out sequencing artifacts and germline events. Strelka2, on the other hand, was run independently using the matched tumor-normal sample pairs to filter out germline variants. Only variants detected by both callers and with a filtering status of PASS were retained for downstream analysis, while variants exclusive to one caller were discarded. The intersection of the two callers was determined by converting the Strelka2 VCF-files to BED-files using BEDOPS *convert2bed* (v2.4.39), and the resulting regions were selected from the Mutect2 VCF-files using VCFtools (v0.1.16)^59,60^. Variant annotation and effect prediction was carried out using snpEff (v4.3t), utilizing a custom-built database derived from canFam4 NCBI annotations^24^. Canine phyloP scores, generated for the reference genome canFam4 and obtained from the publicly available Zoonomia project resource, were mapped to each variant using BEDTools (v2.29.2)^23,61^.

Outlier samples with unusually high or low variant counts were identified using z-scores, with a cutoff of ± 2. These outliers were subsequently excluded from the analysis, resulting in the removal of one sample and a final sample size of 43 individuals.

### Variant selection

Considering this study’s focus on the coding regions of the genome, only variants annotated in protein-coding regions were retained in the final variant dataset. Furthermore, synonymous variants, which are predicted to have no impact on the encoded amino acid sequence, were also eliminated, leaving only non-synonymous coding variants for the subsequent analysis.

### SMGs and RMGs

Significantly Mutated Genes (SMGs) were detected using the dNdScv R package (v.0.0.1.0; RStudio v.4.3.1) with a global *q*-value threshold of ≤ 0.1.^27^ A custom dNdScv reference database for canFam4 was built from the canFam3 Ensembl gene database (v104) which was lifted over to canFam4 coordinates using LiftOver (https://genome.ucsc.edu/cgi-bin/hgLiftOver).

To identify Recurrently Mutated Genes (RMGs), the mutation frequency per gene was calculated, defined as the number of mutated samples divided by the total number of samples, using a threshold of 5% (*n* = 3). Genes with a mutation frequency equal or greater than this threshold were considered RMGs. The RMGs list was then cross-referenced with both the Catalogue Of Somatic Mutations In Cancer (COSMIC) (https://cancer.sanger.ac.uk/cosmic) database and the EpiFactors database (https://epifactors.autosome.org/) to explore potential associations with human cancer and investigate their involvement in epigenetic regulation^34–36^.

### Pathway analysis

All RMGs were input into the g: Profiler web tool (https://biit.cs.ut.ee/gprofiler/gost) in descending order of mutation frequency. Only GO biological processes and Reactome were selected as data sources. Default settings were maintained for all parameters except ‘ordered query’, which was enabled. A significance threshold of *q* ≤ 0.05 was applied for pathway enrichment analysis.

### CNA analysis

Copy Number Aberration (CNA) analysis was conducted using CNVkit (v0.9.10) with the *batch* command^37^. To reduce the number of false positives, bins with low coverage, defined as a read depth of 0 or close to 0, and confidence intervals overlapping 0 were filtered out using the *–drop-low-coverage* and –*filter ci* options. Log2 ratio thresholds for calling copy number changes were set at < -1.1 (deletion), < -0.4 (loss), ≥ 0.3 (gain), and ≥ 0.7 (amplification).

To identify the most significant amplifications and deletions across the entire dog cohort, GISTIC2.0 (v2.0.22)^38^ was used with the following parameters: *-ta 0.2 -td 0.2 -conf 0.95 -genegistic 1 -savegene 1 -armpeel 1 -maxseg 25000 -broad 1 -brlen 0.7 -cap 1.5 -qvt 0.10.*

To enable the use of GISTIC2.0 with data mapped to the canine reference genome canFam4, a custom reference mat file was generated based on the method outlined by Martinez and Amin (https://github.com/sbamin/canine_gistic2).

### Tumor mutational burden

Tumor Mutational Burden (TMB) per sample was calculated as follows: TMB = total number of somatic mutations per sample / total number of bases in the canine reference genome canFam4 in Mb.

### Survival analysis

Progression Free Survival (PFS) was calculated in days from the time of first treatment until the time of disease progression or relapse. The overall survival time (OST) was calculated in days from the time of first treatment until patient’s death. Patients were censored from the analysis if death was due to an unrelated cause (i.e., other underlying disease) or if the patient was still alive at the time of analysis.

First, an explorative univariate Cox proportional hazard regression was performed for each RMG (mutated, wildtype), CNA (CNA, wildtype), and clinical variable including treatment arm (1, 2, 3, 4, 5, 6), sex (female, male), age group (< 8 years, ≥ 8 years), and TMB group (< 1.8, ≥ 1.8) to evaluate their individual associations with PFS and OST. This analysis was performed using RStudio (version 2023.09.0 + 463) and the R packages *survival* and *survminer*. All variables with a *p-*value ≤ 0.10 were selected and included in a multivariate Cox regression model.

### Plots

Lollipop plots were generated using the R package Maftools, and the main oncoplot was created using the Python package CoMut^62,63^.

### Statement on LLM usage

All scientific content, analysis, interpretations, and conclusions presented in this work are original and solely attributed to the authors. The manuscript was entirely written by the authors, with Large Language Model (LLM) usage strictly limited to language enhancement, synonym suggestion, and grammar review.

## Electronic supplementary material

Below is the link to the electronic supplementary material.

Supplementary Material 1

Supplementary Material 2

## Data Availability

The data supporting this study is provided as supplementary information. All raw sequencing data and metadata has been deposited in the European Nucleotide Archive (ENA) at EMBL-EBI under accession number PRJEB77298.
