# Supplementary material for: Characterization of the genomic landscape of canine diffuse large B-cell lymphoma reveals recurrent H3K27M mutations linked to progression-free survival
Source: Sci Rep. 2025 Feb 8;15:4724. doi: 10.1038/s41598-025-89245-0 (PMC11807134; doi:10.1038/s41598-025-89245-0)
Supplement: Supplementary file 2 — Supplementary Material 2 [file 41598_2025_89245_MOESM2_ESM.pdf]

Characterization of the genomic landscape of canine diffuse large B-cell lymphoma reveals recurrent H3K27M mutations linked to progression-free survival

Anna Darlene van der Heiden[1,2*], Raphaela Pensch[1,2], Sophie Agger[4], Heather L. Gardner[6], William Hendricks[3], Victoria Zismann[3], Shukmei Wong[3], Natalia Briones[3], Bryce Turner[3], Karin Forsberg-Nilsson[2,5], Cheryl London[6], Kerstin Lindblad-Toh[1,2,7], Maja Louise Arendt[1,4*]

[1] Department of Medical Biochemistry and Microbiology, Uppsala University, Uppsala, Sweden
[2] SciLifeLab, Uppsala University, Uppsala, Sweden
[3] Division of Integrated Cancer Genomics, Translational Genomics Research Institute (TGen), Phoenix, Arizona, US
[4] Department of Veterinary Clinical Sciences, University of Copenhagen, Copenhagen, Denmark
[5] Department of Immunology, Genetics and Pathology, Uppsala University, Uppsala, Sweden
[6] Cummings School of Veterinary Medicine, Tufts University, North Grafton, Massachusetts, United States of America
[7] Broad Institute of MIT and Harvard, Cambridge, Massachusetts, United States of America

*anna.vd.heiden@imbim.uu.se
*maja.arendt@sund.ku.dk

Supplementary Figure S1

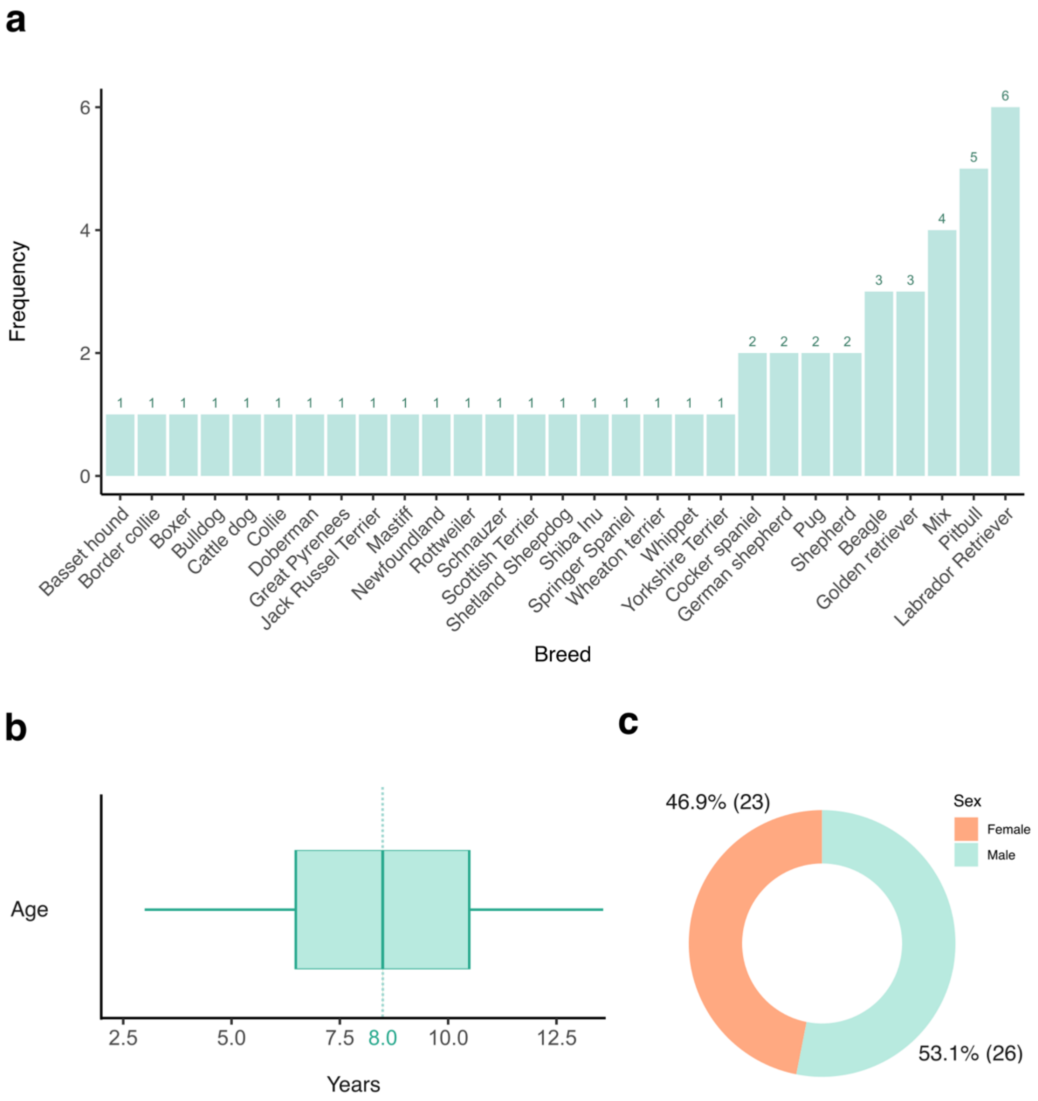

Figure S1. Breed distribution, age, and sex of canine samples in our study cohort. **a)** Breed distribution of dogs included in the study. **b)** Age of patients at time of diagnosis. The median age is denoted by a dashed vertical line. **c)** Sex distribution of canine patients (*n* = 23 females, *n* = 26 males).

Supplementary Figure S2

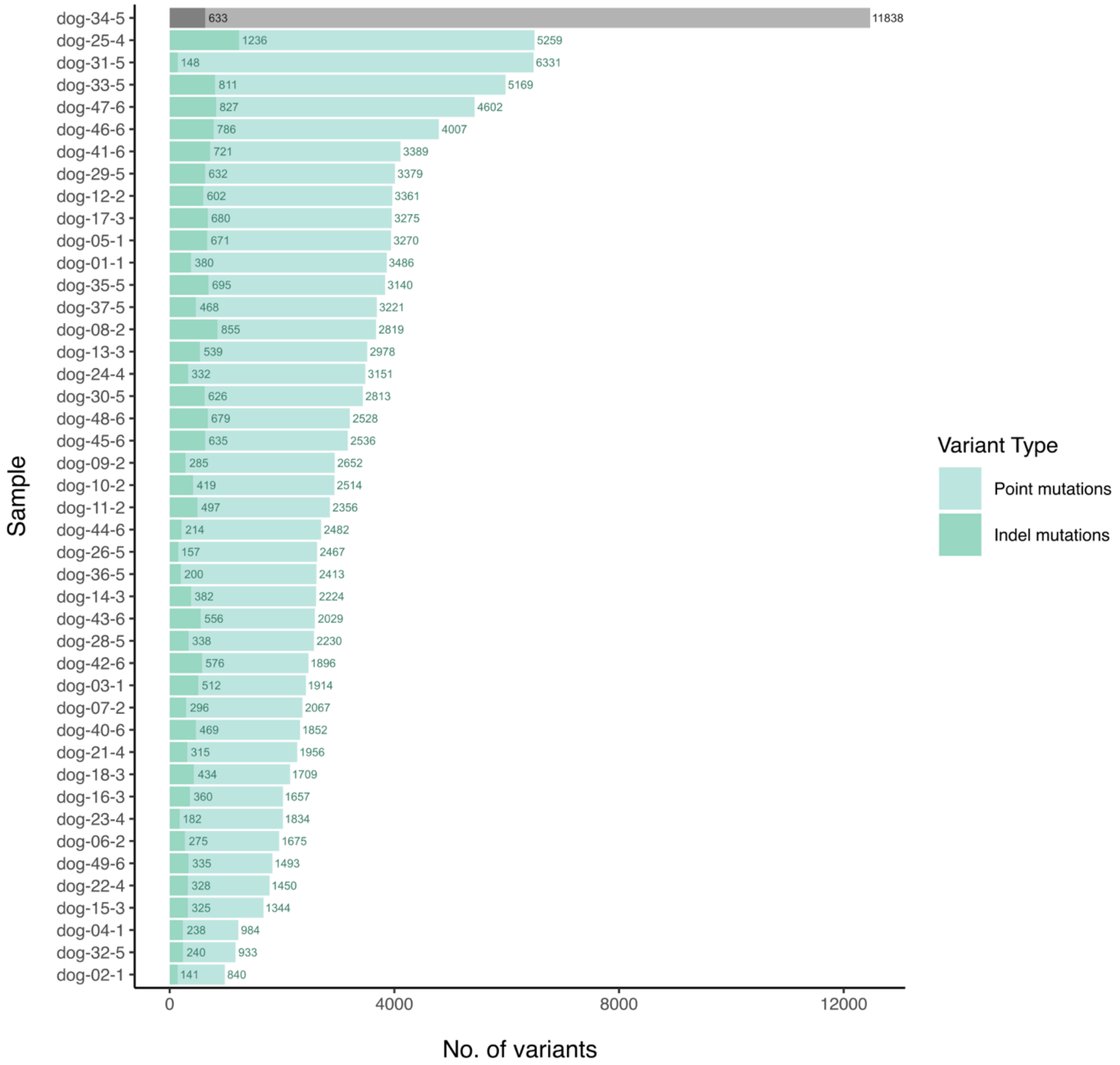

Figure S2. Somatic variant count per sample. Point mutations are shown in light blue, while indels are shown in green. Samples with a z-score ≥ 2 (highlighted in grey) were considered outliers and excluded from the analysis.

Supplementary Figure S3

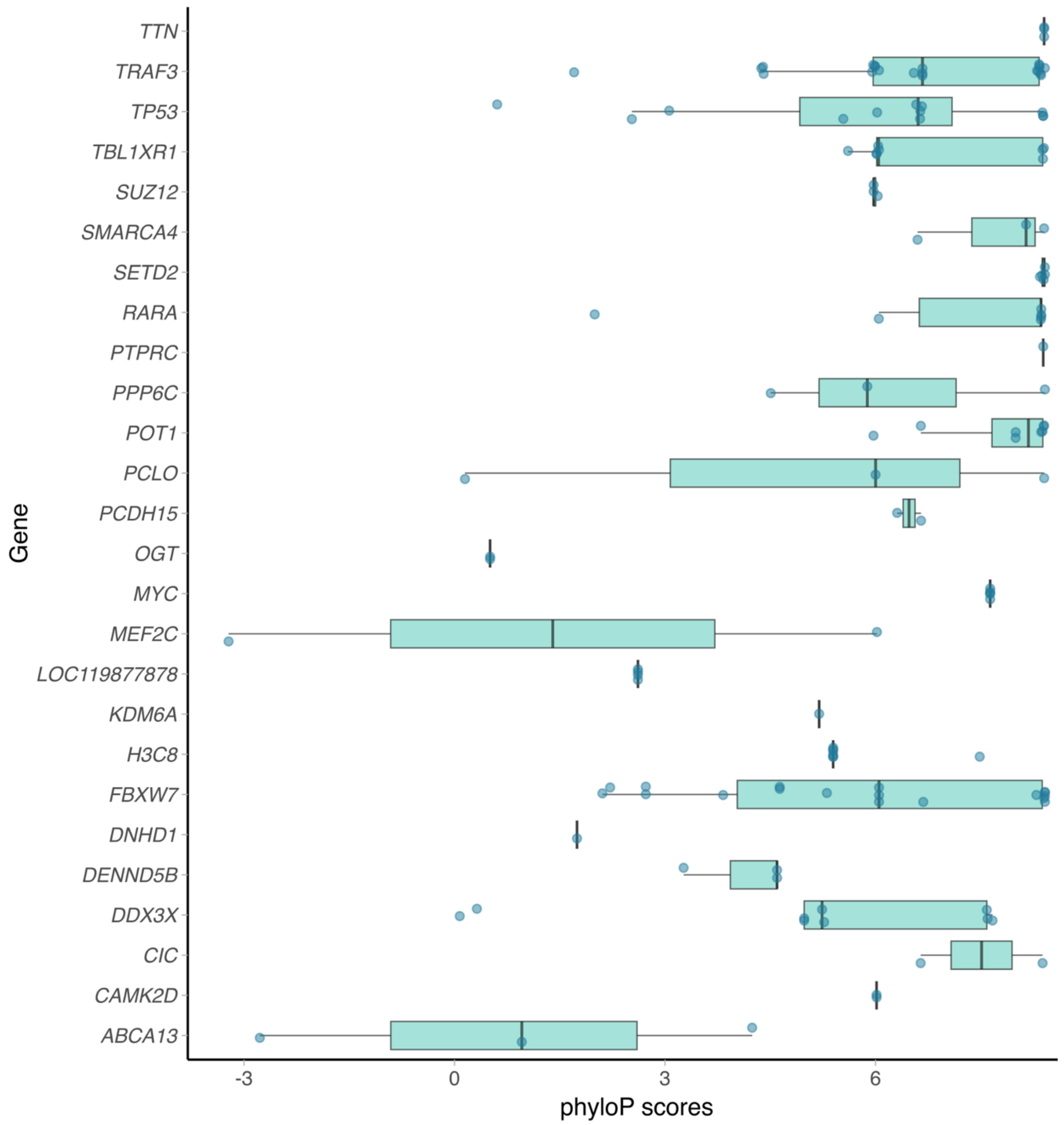

Figure S3. Distribution of point mutations in recurrently mutated genes, arranged by phyloP conservation scores.

Supplementary Figure S4

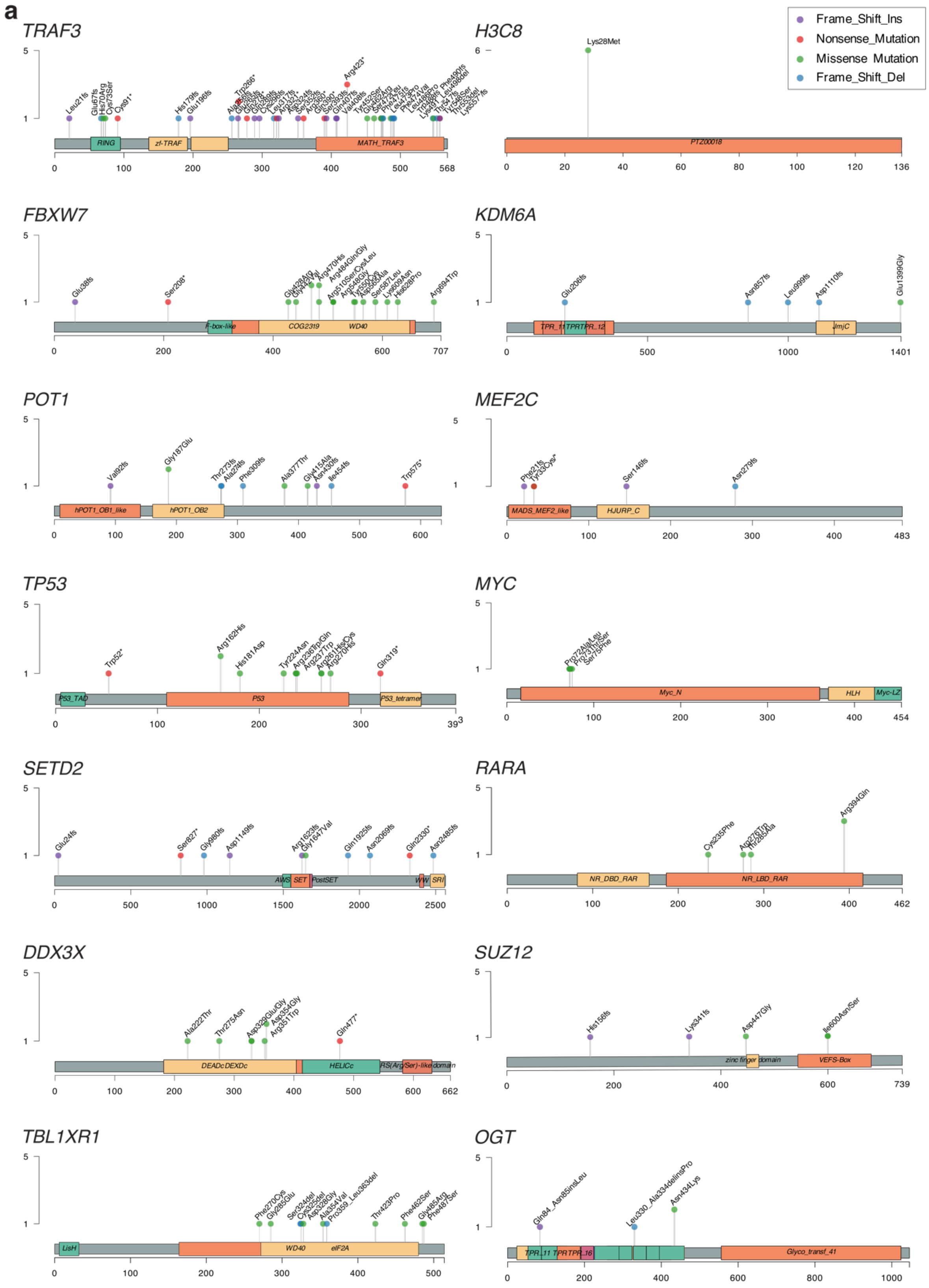

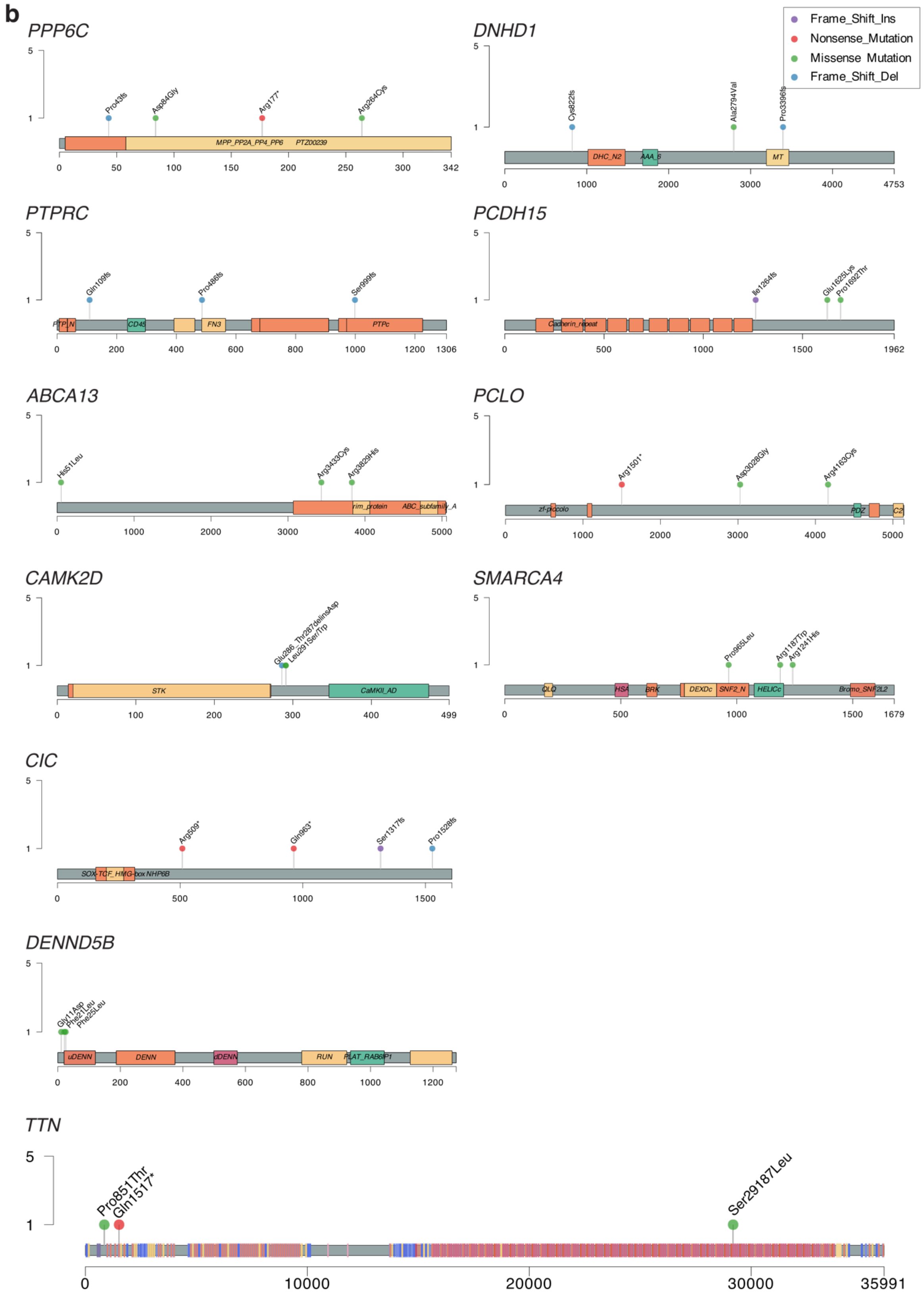

Figure S4. Lollipop plots showcasing all nonsynonymous mutations identified in each Recurrently Mutated Gene (RMG). The gene *LOC119877878* is excluded because MAFtools could not generate a plot due to insufficient annotation. **a)** First 14 RMGs, arranged in descending order of mutation frequency (starting at top left, ending at bottom right). **b)** Remaining 11 RMGs.

Supplementary Figure S5

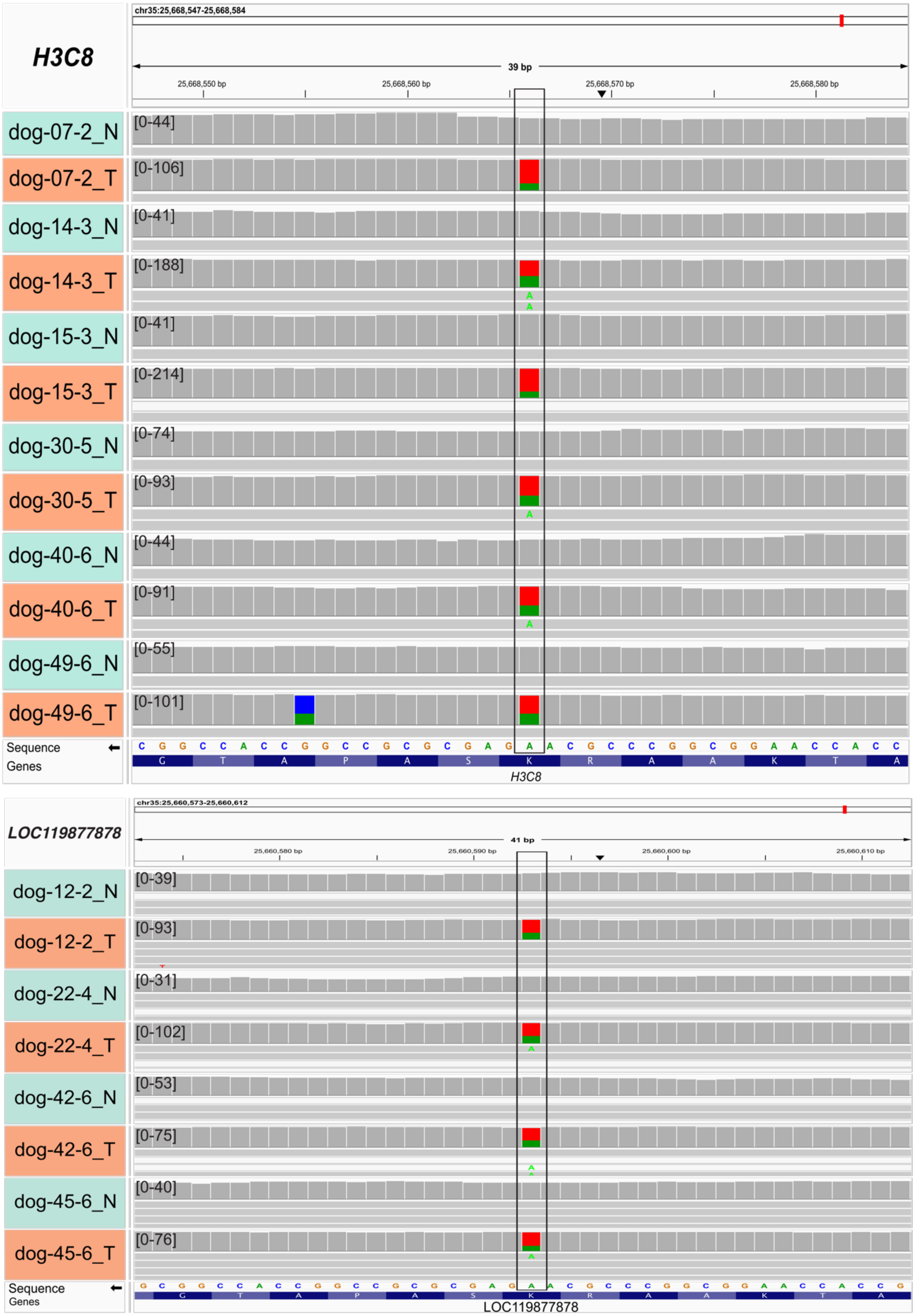

Figure S5. Integrative Genomics Viewer (IGV) snapshot of the top two recurrently mutated base pair positions in the cohort. The tumor samples are highlighted in orange, while the matched normal samples are shown in blue.

Supplementary Figure S6

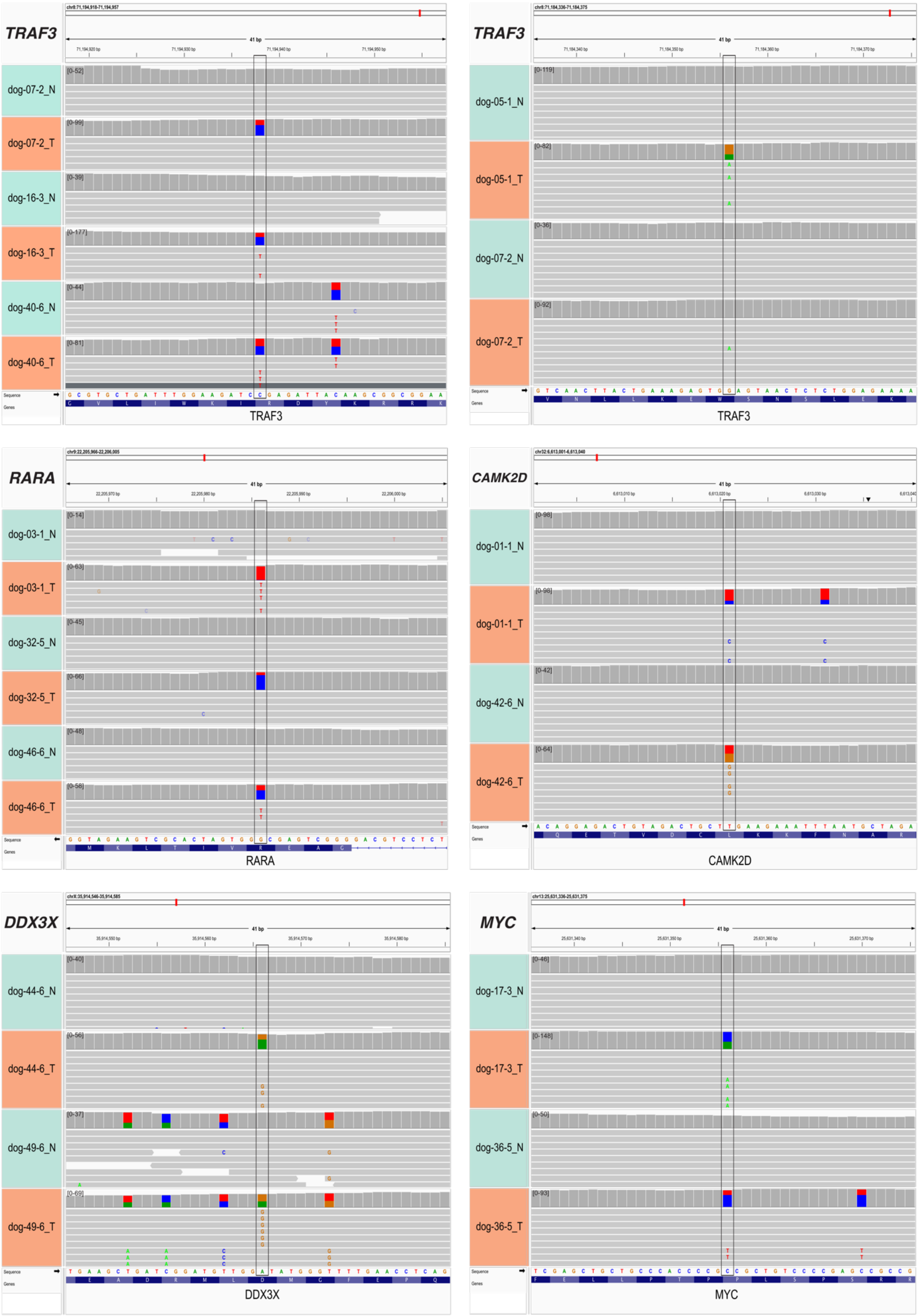

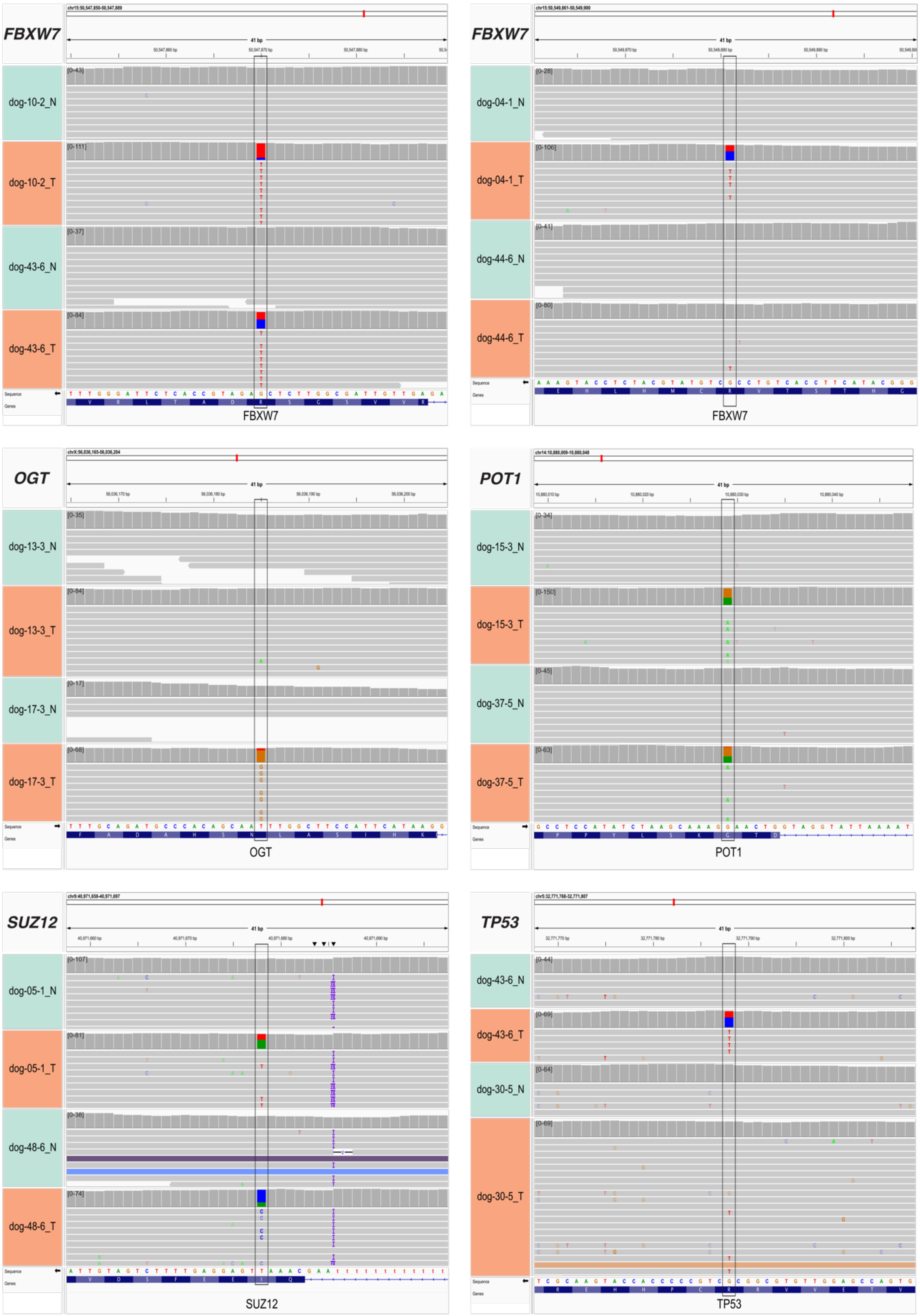

Figure S6. Integrative Genomics Viewer (IGV) snapshot of the remaining recurrently mutated base pair positions found in the cohort. The tumor samples are highlighted in orange, while the matched normal samples are shown in blue.

Supplementary Figure S7

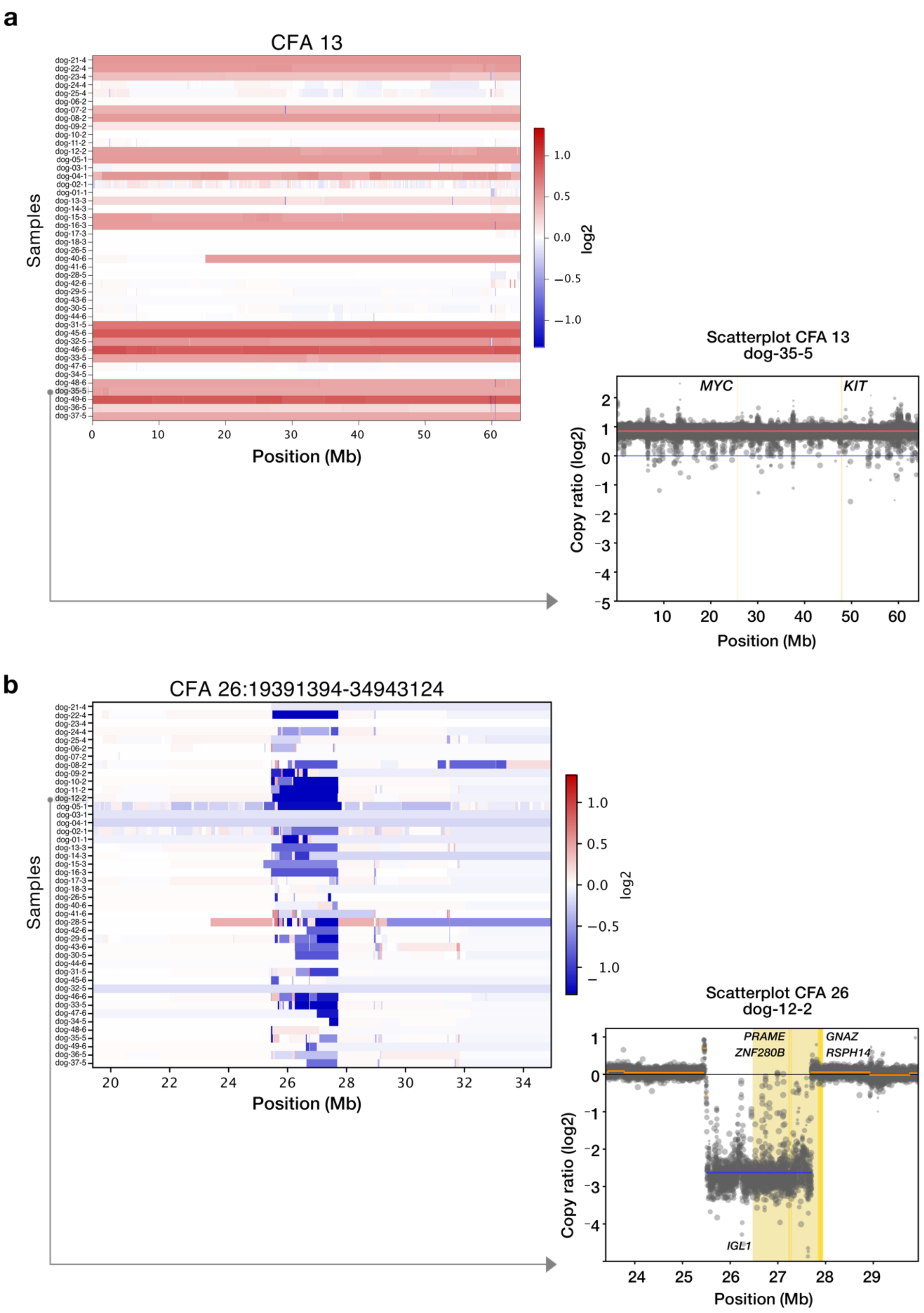

Figure S7. Top Copy Number Alterations (CNAs). **a)** Left: Heatmap of top amplificatioin, showing a broad amplificatioin of CFA 13. Right: Scatter plot of the amplification in sample dog-35-5, generated using CNVkit's *scatter* command. The plot displays bin-level log2 coverages and segmentation calls. The red line represents the trend, while yellow highlights indicate the positions of cancer genes *MYC* and *KIT.* **b)** Left: Heatmap of top deletion, showing a focal deletion in CFA 26. Right: Scatter plot of the deletion in sample dog-12-2. In this case, the overall trend is shown with an orange line, while the deletion trend is represented by a blue line. Yellow highlights indicate the positions of genes *PRAME, ZNF280B, GNAZ, RSPH14,* and *IGL1* (larger section in light yellow).
